# Long-Term Follow-Up of Classical Hodgkin Lymphoma and Diffuse Large B-Cell Lymphoma Survivors: Aims and Methodological Approach for Fondazione Italiana Linfomi Systematic Reviews

**DOI:** 10.3390/cancers13122976

**Published:** 2021-06-14

**Authors:** Chiara Gerardi, Eleonora Allocati, Carla Minoia, Attilio Guarini, Rita Banzi

**Affiliations:** 1 Istituto di Ricerche Farmacologiche “Mario Negri” IRCCS, 20156 Milano, Italy; eleonora.allocati@marionegri.it (E.A.); rita.banzi@marionegri.it (R.B.); 2 Hematology Unit-IRCCS Istituto Tumori “Giovanni Paolo II”, 70124 Bari, Italy; c.minoia@oncologico.bari.it (C.M.); attilioguarini@oncologico.bari.it (A.G.)

**Keywords:** cancer survivorship, Hodgkin lymphoma, diffuse large B-cell lymphoma, follow-up, systematic review

## Abstract

**Simple Summary:**

In 2019, the Fondazione Italiana Linfomi research team proposed a new project to summarize the evidence on late sequelae after treatment of classical Hodgkin lymphoma and diffuse large B cell lymphoma in long-term survivors (>5 years free of disease). Six systematic reviews were conducted. Clinical research questions focused on the incidence of late toxicities, comparison of toxicities with different chemotherapies, radiotherapies and autologous stem-cell transplantation, and evidence on early detection and long-term follow-up of late sequelae. The six reviews investigated the late impact of treatment on cardiological diseases, secondary neoplasms, metabolic and endocrine, neurological and psychological disorders, preservation of fertility and reproductive status, and the effect of lifestyle and nutrition. The literature search was conducted on Pubmed, Embase, and Cochrane Library databases up to 2020. This project summarized current evidence in order to see which course of action was best applicable for the clinical management of long-term survivors after Hodgkin lymphoma and diffuse large B cell lymphoma, and highlighted the gaps in the field.

**Abstract:**

Advances in diagnosis and treatment of hematological malignancies has boosted attention on optimal follow-up care of survivors after cancer. To collect evidence that could inform the development of an optimal model for Italian hematology centers and the scientific community, Fondazione Italiana Linfomi (FIL) commissioned an analysis of the international follow-up approaches for long-term survivors after classical Hodgkin lymphoma (cHL) or diffuse large B-cell lymphoma (DLBCL). FIL set up multidisciplinary teams, representing all different skills relevant for cancer survivors. They conducted a series of systematic reviews focused on three main aspects: incidence of long-term toxicity; comparison of old or standard therapies and more recent ones; and evidence on specific follow-up approaches. The teams applied this framework to cardiological, endocrine-metabolic, neurological/cognitive, and psychological disorders, secondary cancers, fertility preservation, and lifestyles. Each team conducted comprehensive literature searches on PubMed, Embase and Cochrane Library databases up to 2020. Screening followed the PRISMA statement for reporting systematic reviews. The reviews report the results of this wide project covering the main areas of late toxicity and conditions in the long-term survival of cHL and DLBCL patients and their follow-up. From a clinical point of view, the series confirmed that the evidence on follow-up tended to focus on solid tumors with scant evidence on hematological malignancies.

## 1. Introduction

Advances in the diagnosis and treatment of hematological malignancies has boosted attention on the optimal follow-up care of long-term survivors. International medical societies and health authorities have developed follow-up programs to support patients after complete remission to improve quality of life (QOL) and monitor long-term sequelae of cancer treatments. Europe’s Beating Cancer Plan, launched by the European Commission in early 2020, focused on follow-up care, in addition to prevention, early diagnosis, and treatment [1].

The essential components of survivorship care include prevention and surveillance of new tumors or relapses of primary diseases and long-term sequelae, as well as the management of the effects of therapies. Survivorship care requires the collaboration of specialists and primary care providers, ideally in multidisciplinary teams. Several out-patient models have been developed: multidisciplinary survivorship clinics, as for instance those dedicated to the long-term follow up of cancers developed in childhood or adolescence; disease-specific survivor clinics, focused on a given disease, for instance, breast or colorectal cancer; and general survivorship clinics, providing services for a broad spectrum of diseases [2,3].

To manage future health care needs, cancer survivors require a structured survivorship care plan (SCP). Several organizations who coordinate oncological care have developed them–for instance, the American Society of Clinical Oncology [3]. SCPs often include information on treatments received by the patient, the need for future exams and assessments to monitor potential long-term late sequelae of the disease and therapies, and measures for improving health [4].

In 2013, the UK National Health Service (NHS) promoted a document called “Living with and beyond cancer: Taking action to improve outcomes is intended to inform the direction of survivorship work in England to 2015” [5]. It was designed to identify national and local priorities and identify unmet needs to help improve the condition of all patients living with cancer and other ongoing health issues. The survivorship section of this document concluded that ample information is needed on the treatment and care of cancer patients, their QOL, and the support and facilities they require. The document suggested different approaches and best practices, with recommendations for next improvements [5].

Most of the research and clinical experience on the management of cancer survivors come from breast cancer and childhood leukemia. In recent years, however, interest in hematological malignancies has grown. For instance, an SCP was applied to lymphoma patients at the Cancer Free Clinic of Mayo Clinic-Rochester Hospital [6]. From November 2013 to May 2015, an SCP designed to improve lifestyle habits (healthy diet and physical exercise) was tested in 96 patients. Patients who followed the plan were more likely to “definitely” call to mind discussions about health improvement, preventing disease, and making modification in lifestyle/habits, nutrition, and physical activity. The study did not report differences in QOL or distress.

One review described updated evidence on survivorship care models, identifying current gaps [7]. The authors found nine publications regarding programs addressing healthcare needs of survivors after cancer. They identified the four components of care for survivorship: prevention, surveillance, intervention, and coordination, and the model characteristics that may impact on the delivery of care and the outcomes of survivors. Three studies analyzed the differences in SCPs led by physicians, two assessed nurse-led models, three presented the development of SCPs as crucial components, and only one compared individual versus group-based counseling and workouts for survivors.

Wide heterogeneity emerged between the studies, considering the components of the survivorship care models, the type of neoplasm for which survivors had been treated, and the length of the follow-up.

Data from survivorship care models are still lacking, particularly the advantages of different models, the impact on survivors’ health outcomes, barriers to offering survivorship care, assessment of available programs, and the costs and benefits of this model [7]. To help the assessment of SCPs, future studies should adequately describe the models examined and assess their application to the general population. In addition, the collection of information on differences in survivorship care needs among individuals from under-served settings would be important to inform and promote culturally sensitive models [7].

Interesting experiences focused on lymphoma survivors included the nurse-led lymphoma survivorship clinic with a tailored SCP [8,9], and the initiative promoted by the US National Institute of Health on long-term care after hematopoietic cell transplantation (HCT). The latter aimed to define the main issues, considering the specificity of survivorship after transplant. It highlighted research priorities to be addressed by basic research and observational and clinical studies to improve knowledge and understanding of the late effects in this population [10].

In Italy, there is a growing number of out-patient cancer programs focused on lymphoma survivors. The first started in the 1990s at the Centro di Riferimento Oncologico in Aviano and the Humanitas Research Hospital in Milan. A recent survey mapping the current follow-up of the growing numbers of Italian lymphoma survivors showed broad heterogeneity and several multidisciplinary approaches [11].

To collect the evidence to inform the development of an optimal model for Italian hematology centers, the Fondazione Italiana Linfomi (FIL) commissioned an analysis of the international follow-up approaches for long-term survivors after classical Hodgkin lymphoma (cHL) or diffuse large B-cell lymphoma (DLBCL). These two cohorts form the prevalent population of long-term lymphoma survivors.

The specific aims of the proposed series of systematic reviews were to understand the best applicable model for the clinical management of long-term survivors after cHL or DLBCL, highlight the gaps in long-term monitoring and follow-up, and provide suggestions on further research in the field. The overall goal of this project is to help decision-makers and clinicians to plan follow-ups for lymphoma survivors in the near future.

## 2. Materials and Methods

### 2.1. Shaping the Idea, Project Set-Up, and Training

FIL launched its project in 2019. The first step involved setting up a multidisciplinary research team, representing all the different expertise relevant for the comprehensive management of long-term lymphoma survivors. The final research team comprised eight onco-hematologists, two cardiologists, two radiotherapists, a gynecologist, an endocrinologist, a psychologist, and a nutritional biologist. These researchers were part of one or more review teams and were supported by a methods team, comprising three experts in clinical research methodology and systematic reviews (Istituto Di Ricerche Farmacologiche “Mario Negri” IRCCS, Milan).

The research group had a brainstorm meeting in March 2019 to define the clinical areas of interest and the research questions to be addressed by each review team. Six areas were identified: cardiological, endocrine-metabolic, neurological/cognitive and psychological disorders, secondary cancers, fertility preservation, lifestyles, and tailored survivorship care plans. The focus of the project was limited to cHL or DLBCL survivors treated as adults and in remission for more than five years from first- or second-line treatments, including autologous hematopoietic stem cell transplant.

The meeting included the presentation of the methodological approach and a training session for the review teams. The proposal was to conduct a series of systematic reviews, one for each area, sharing a common structure but flexible enough to account for the specifics of the different clinical settings. First, evidence was collected on late effects after treatments in order to understand their incidence in relation to the previous therapeutic approaches. Second, the best approaches to monitoring any long -term sequalae related to toxicity of previous treatments were identified. The review teams were trained on the basic elements of a systematic review according to the Cochrane approach [12] which included clear definitions of the review questions, a systematic literature search, transparent screening and inclusion of studies, and an assessment of the risk of bias. They were also trained on how to report a systematic review following the PRISMA statement [13].

### 2.2. Clinical Questions, PICOS and Identification of Evidence

The reviews were structured around three aspects: (i) the incidence of specific long-term toxicity; (ii) the comparison of old or standard therapies (for which more data are available) and more recent therapies (including modern radiation therapy); (iii) the best method for early detection and monitoring of long-term sequelae.

This framework was applied with minimal adaptations by the teams on cardiological, endocrine-metabolic, neurological/cognitive disorders, and secondary cancers. The groups focusing on fertility preservation and lifestyles and tailored survivorship care plans took a different approach, as the objective of their analysis was slightly different.

Each review team defined the relevant clinical questions and divided them into researchable questions through the population, intervention, comparison, outcome study design (PICOS) framework. This lays the basis for the definition of the inclusion and exclusion criteria of the reviews and for literature searches. Considering the aim of the project, the review team agreed on the inclusion of clinical trials, controlled observational studies (prospective and retrospective), and systematic reviews.

With the support of the methods team, each review team conducted comprehensive literature searches on the following scientific databases: PubMed, Embase, Cochrane Library. Clinical study registries such as Clinicaltrial.gov, and hand-searched references of these studies were done to maximize the sensitivity of the searches. Titles and abstracts were screened by at least two independent reviewers, and the selected full-text publication was examined to confirm eligibility. Discrepancies were resolved by discussion with a third reviewer or a member of another review team. The screening process was reported using the PRISMA flow-chart [13].

### 2.3. Evidence Synthesis and Appraisal, Drawing Conclusions

A study was considered eligible if information pertinent to one or more PICOS item was abstracted in a tabular format. The key elements can be summarized as: study design, duration, population, sample size, interventions, comparators, follow-up, outcome measures, main results and main conclusions, and reference. The evidence was summarized qualitatively as a meta-analysis was not considered adequate in this setting. Each review team assessed the risk of bias in randomized trials using the Cochrane risk of bias tool v1 [14], the quality of observational studies with the New Castle Ottawa Scale [15], and the quality of systematic reviews with AMSTAR 2 [16]. Appropriate critical appraisal of evidence is a key component of a well-conducted systematic review, as the credibility of evidence syntheses can be compromised if biases are not adequately highlighted and taken into consideration when drawing conclusions. All these steps were done by the review teams with the constant support of the methods team. After data extraction, each group wrote a report on the disease area and discussed the findings in light of current knowledge, including guidelines and recommendations of the major scientific societies.

## 3. Results and Discussion

To our knowledge, this is the first comprehensive assembly of evidence on clinical management of long-term survivors after cHL or DLBCL. We hope our results will support guideline development and prioritization of research funding allocation, providing the evidence base on which recommendations may be developed, following a transparent and robust methodology. Specifically, it should inform the definition of optimal programs to support patients after complete remission of lymphoma and highlight the gaps where specific research programs are now important. The initiative started with bottom-up input from health professionals involved in the care of long-term lymphoma survivorship, who acknowledged a research priority and set up a project to respond to specific issues. The integration of different competences in a joint, coordinated effort is a major achievement in view of the multidisciplinary approach needed for long-term survivors. The project highlighted several gaps still not covered that could be taken into consideration in new research projects promoted by FIL or other funders (such as scientific societies).

The following series reports the results of a wide project covering the main areas of late toxicity and conditions in the long-term survival of cHL and DLBCL patients and their follow-ups.

While each article in the series will report detailed considerations, some main clinical and methodological conclusions can be drawn from an analysis of the evidence.

From a clinical point of view, our series confirmed that the evidence on follow-up pathways focused mainly on solid tumors with scant information on hematological malignancies. cHL and DLBCL patients can be considered a separate population from those with other hematological diseases due to their good prognosis and life expectancy, the novel chemo-immunotherapy and PET/CT-guided approach during chemo-radiotherapy for patients with cHL, and the improved diagnosis of DLBCL [17].

The improved survival of these patients calls for tailored follow-up specific to their characteristics based on treatments received and their disease history. This is in line with guidelines on solid tumors, such as the National Comprehensive Cancer Network (NCCN) for survivorship and the European Society for Medical Oncology (ESMO) guidelines for monitoring cardiac toxicity in survivorship [18,19]. Follow-up should also be tailored on the basis of treatments received (dose and regimen), age at diagnosis, and baseline risk factors. New follow-up pathways should involve different figures and be integrated into hospital-primary care programs. Hematologists and clinicians with specific knowledge on disease management and long-term risk factors should work closely with primary care providers, such as general practitioners—who have a comprehensive view of their patients and can provide valuable care and relevant information.

A recent analysis reviewed cancer survivorship models, with a particular focus on those aimed at integrating primary care providers into the care of cancer survivors [20]. Although several models include primary care providers, the descriptions and evaluation of different approaches are limited. Health professionals such as onco-generalists—primary care providers with expertise in survivorship who can merge the composite needs of cancer survivors—emerged as experts in the care of cancer survivors in specific centers and academic or community hospitals, and as possible links between primary care and oncology providers. This model may lend itself to the care of survivors who are at lower risk of cancer-related complications. The take-home messages of this review include a call for researchers and policy-makers to improve their efforts at creating and implementing evidence-based guidelines and evaluate their quality of care [20].

Like other surveillance programs, the management of long-term lymphoma survivors must assess the potential overuse of diagnostic procedures or treatments. The international initiative Choosing Wisely reports a generic recommendation to start surveillance testing after cancer treatment only after providing the patient with a survivorship care plan to help in transitioning to long-term surveillance, avoiding unnecessary services, and seeking appropriate care and decision support [21]. Choosing Wisely also assessed the use of computer tomography surveillance in adults in long-term remission after DLBCL. The study, conducted in Canada using a population-based administrative database from Ontario, highlighted the excessive use of surveillance imaging during a timeframe in which it is deemed unnecessary [22].

The overuse of diagnostic procedures or treatments may be associated with side effects, including the possible psychological distress caused by the status of being a “cancer survivor”. In the broader context of a patient-centered perspective on cancer, the development and implementation of survivorship plans should include a transparent approach to shared decision making, where patients’ preferences are taken into consideration.

From a methodological point of view, our series pointed out two main limitations. The first regards the indirectness of the results. Most of the evidence refers to mixed populations that include different hematological tumors and, in some cases, even solid tumors, so a good number of studies had to be excluded. The lack of stratification between solid and hematological malignancies and thorough evaluation of the differences precludes robust identification of the most adequate features in each setting. When trials and observational studies only assessed lymphoma survivors, they usually included groups of participants with different disease histories. In general, there is a lack of data on DLBCL patients and those who utilize second-line treatments such as transplants for both cHL and DLBCL. Other papers reported data on populations including children as well as adults, or mixed long-term survivors and patients still receiving treatment together without stratification. The study populations in almost all the studies varied as regards treatment received, age at diagnosis, and timing of the survivorship (intended as time after diagnosis).

The second issue regards the overall quality of the evidence. Studies were seldom sufficiently powered to detect long-term sequalae as primary or secondary outcomes, and follow-up was generally too short to detect relevant medical issues in prospective observational and randomized controlled trials. Studies often had major methodological flaws and employed inadequate comparators.

## 4. Conclusions

This project, promoted by FIL, summarized current evidence to find the best model for the clinical management of long-term survivors after cHL or DLBCL and offered suggestions on further research in the field. It also built up a new multidisciplinary research group, including hematologists with other relevant figures such as experts in radiotherapy, endocrinology, nutrition, and psychology, and research methodology. This group could support decision-makers in preparing sustainable evidence-based guidelines such as SCP, on an Italian national level, and help to fill research gaps through new projects covering unmet medical needs for lymphoma patients.

## Data Availability

Not applicable.

## References

[1] EU Policy on Cancer, Cancer, European Commission. https://ec.europa.eu/health/non_communicable_diseases/cancer_en.

[2] Hewitt M., Weiner S.L., Simone J.V., Hewitt M., Weiner S.L., Simone J.V. (2003). Childhood Cancer Survivorship: Improving Care and Quality of Life.

[3] Council IoMaNR (2006). National Research Council 2006. From Cancer Patient to Cancer Survivor: Lost in Transition.

[4] ASCO Prevention & Survivorship -Survivorship Care Planning Tools. https://www.asco.org/practice-policy/cancer-care-initiatives/prevention-survivorship/survivorship-compendium.

[5] DH, Macmillan Cancer Support & NHS Improvement Living with & Beyond Cancer: Taking Action to Improve Outcomes (An Update to the 2010 The National Cancer Survivorship Initiative Vision). https://assets.publishing.service.gov.uk/government/uploads/system/uploads/attachment_data/file/181054/9333-TSO-2900664-NCSI_Report_FINAL.pdf.

[6] Viscuse P., Yost K.J., Jenkins S., Lackore K., Habermann T., Thanarajasingam G., Thompson C. (2019). Impact of lymphoma survivorship clinic visit on patient-centered outcomes. J. Cancer Surviv..

[7] Halpern M.T., Viswanathan M., Evans T.S., Birken S.A., Basch E., Mayer D.K. (2015). Models of Cancer Survivorship Care: Overview and Summary of Current Evidence. J. Oncol. Pract..

[8] Taylor K., Joske D., Bulsara M., Bulsara C., Monterosso L. (2016). Protocol for Care After Lymphoma (CALy) trial: A phase II pilot randomised controlled trial of a lymphoma nurse-led model of survivorship care. BMJ Open.

[9] John C., Armes J. (2013). Developing a nurse-led survivorship service for patients with lymphoma. Eur. J. Oncol. Nurs..

[10] Battiwalla M., Tichelli A., Majhail N.S. (2017). Long-Term Survivorship after Hematopoietic Cell Transplantation: Roadmap for Research and Care. Biol. Blood Marrow Transplant..

[11] Minoia C., Bari A., Nassi L., Banzi R., Gerardi C., Lenti V., Calabrese M., Spina M., Guarini A. (2021). Management of lymphoma survivor patients in Italy: An evaluation by Fondazione Italiana Linfomi. Tumori J..

[12] Higgins J.P.T., Thomas J., Chandler J., Cumpston M., Li T., Page M.J., Welch V.A. (2019). Cochrane Handbook for Systematic Reviews of Interventions.

[13] Moher D., Liberati A., Tetzlaff J., Altman D.G., Group P. (2009). Preferred reporting items for systematic reviews and meta-analyses: The PRISMA statement. PLoS Med..

[14] Higgins J.P., Altman D.G., Higgins J.P., Green S. (2010). The Cochrane Collaboration’s tool for assessing risk of bias. Cochrane Handbook for Systematic Reviews of Interventions.

[15] Wells G.A., Shea B., O’Connell D., Peterson J., Welch V., Losos M., Tugwell P. (2011). The Newcastle-Ottawa Scale (NOS) for Assessing the Quality of Nonrandomised Studies in Meta-Analyses.

[16] Shea B.J., Reeves B.C., Wells G., Thuku M., Hamel C., Moran J., Moher D., Tugwell P., Welch V., Kristjansson E. (2017). AMSTAR 2: A critical appraisal tool for systematic reviews that include randomised or non-randomised studies of healthcare interventions, or both. BMJ.

[17] National Comprehensive Cancer Network Guidelines Version 2.2020 “Survivorship”. NCCN GUIDELINES. https://www.nccn.org/professionals/physician_gls/pdf/survivorship.pdf.

[18] Haematological Malignancy Research Network (HMRN). https://www.hmrn.org/statistics/survival.

[19] Curigliano G., Lenihan D., Fradley M., Ganatra S., Barac A., Blaes A., Herrmann J., Porter C., Lyon A., Lancellotti P. (2020). Management of cardiac disease in cancer patients throughout oncological treatment: ESMO consensus recommendations. Ann. Oncol..

[20] Nekhlyudov L., O’Malley D.M., Hudson S.V. (2017). Integrating primary care providers in the care of cancer survivors: Gaps in evidence and future opportunities. Lancet Oncol..

[21] Choosing Wisely Commission on Cancer. https://www.choosingwisely.org/societies/commission-on-cancer/.

[22] Cheung M.C., Mittmann N., Earle C.C., Rahman F., Liu N., Singh S. (2018). Are We Choosing Wisely in Lymphoma? Excessive Use of Surveillance CT Imaging in Patients With Diffuse Large B-cell Lymphoma (DLBCL) in Long-term Remission. Clin. Lymphoma Myeloma Leuk..

